# Wainwright’s Urinary Diagnosis

**Published:** 1902-10

**Authors:** 


WAINWRIGHT'S URINARY DIAGNOSIS. By J. W. Wainwright, M. D., Member of the American Medical Association, New York State Medical Association, New York County Medical Association, etc. Illustrated with numerons engravings and colored plates. Pages, 140. Price, $1 net.

This book gives not only all the usual methods of urinary examination, but introduces also a new feature in works of this character, viz., a discussion of the clinical significance of the urinary findings and their practical application in treating the diseases of which they are symptomatic.

Among the subjects discussed are the following: Composition and Physical Character of the Urine; Normal Constituents of Urine; Abnormal Constituents of Urine; the Microscope and Microscopical Technique; Qualitative Analysis or Urinary Calculi; Bright's Disease, Diabetes, Gout and Other Conditions and Their Treatment; Favorite Prescriptions, etc. T. J. B.

